# Ion-Ion Proton Transfer and Parallel Ion Parking for the Analysis of Mixtures of Intact Proteins on a Modified Orbitrap Mass Analyzer

**DOI:** 10.1007/s13361-019-02290-8

**Published:** 2019-08-07

**Authors:** Scott A. Ugrin, A. Michelle English, John E. P. Syka, Dina L. Bai, Lissa C. Anderson, Jeffrey Shabanowitz, Donald F. Hunt

**Affiliations:** 1 Department of Chemistry, University of Virginia, Charlottesville, VA 22904 USA; 2 Thermo Fisher Scientific, San Jose, CA 95134 USA; 3 Ion Cyclotron Resonance Program, National High Magnetic Field Laboratory, Tallahassee, FL 32310 USA; 4 Department of Pathology, University of Virginia, Charlottesville, VA 22908 USA

**Keywords:** Quadrupole linear ion trap, Ion-ion proton transfer reactions, Parallel ion parking, Reagent activation, Intact protein, Top-down

## Abstract

We have enabled parallel ion parking on a modified Orbitrap Elite™ as a way to control ion-ion proton transfer reactions via selective activation of a range of ions. The result is the concentration of the majority of ion current from multiple charge states of each precursor proteoform into a single charge state, maximizing signal intensity and increasing effective sensitivity compared to conventional MS1 spectra. These techniques were applied in an on-line HPLC, data-dependent MS/MS analysis of intact *E. coli* ribosomal proteins with HCD fragmentation. With one injection, all but two ribosomal proteins were selected for fragmentation and subsequently identified. The techniques described facilitate rapid identification of intact proteins in complex mixtures and an enhanced ability to observe proteins of low abundance.

**Electronic supplementary material:**

The online version of this article (10.1007/s13361-019-02290-8) contains supplementary material, which is available to authorized users.

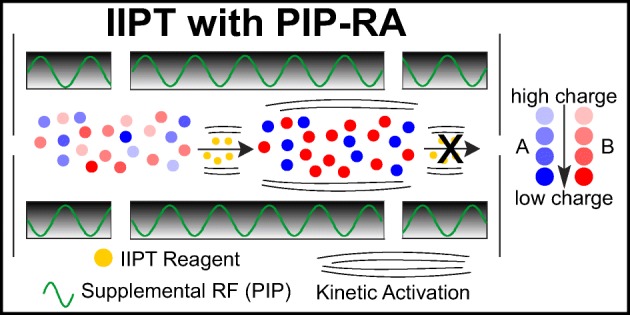

## Introduction

Studies involving mass spectrometric analysis of intact proteins in complex mixtures are subject to many analytical challenges owing to the low abundance of many of these proteins and to the complexity of the samples analyzed. These challenges are simultaneously mitigated and compounded by use of electrospray ionization [1] (ESI), which enables direct coupling to HPLC to reduce sample complexity, but also ionizes each protein analyte to multiple charge states and divides the signal among several m/z peak isotopic envelopes [2]. The latter effect is magnified as the electrosprayed proteins grow in size, especially if electrospray is performed under denaturing conditions [3]. Additionally, while there have been improvements in on- and offline separation methods for intact protein analysis [4–9], the resolution of intact protein separations by reverse-phase chromatography is often not high enough to avoid extensive co-elution of proteins [10, 11]. These complications coupled with the limited charge capacity of the high resolution ion trapping mass spectrometers primarily used for such work, translate to significantly lower signal-to-noise ratios (S:N) in comparison to corresponding peptide-based proteomics experiments.

Over two decades ago, Stephenson and McLuckey introduced ion-ion proton transfer (IIPT) chemistry for simplification of mass spectra derived from multiply charged precursor ions [12]. IIPT involves use of reagent anions that function as Brønstead bases, abstracting protons from multiply charged precursors. The rate of an IIPT reaction is directly proportional to the square of the charge states of the analyte(s) and reagent (usually singly charged) ions [13]. If the reaction is allowed to proceed in an uncontrolled manner, products of a single IIPT reaction [M+(n-1)H]^(n-1)^ will continue to react due to continued exposure to reagent anions. Thus, absent special measures, exposure of an initial population of highly charged (typical for intact protein ions produced by positive ESI in non-native conditions), m/z-selected protein precursor ions (Supplemental Figure 1a) to a substantially greater population of IIPT reagent ions for some given time interval will produce a multiplicity of intact charge-reduced protein product ions that are the result of variable numbers of successive IIPT reactions (Supplemental Figure 1b). Extending IIPT reaction (exposure) times results in a lower mean charge state of the product ion charge state distribution (Supplemental Figure 1 b-c). Continued IIPT reactions will eventually generate charge-reduced products that fall outside of the available m/z range of typical mass analyzers (Supplemental Figure 1 d-e). Fortunately, the McLuckey group developed methods termed “ion parking” [14] and “parallel ion parking” (here referred to as PIP) [15, 16]. Here, we focus on PIP, a method of selectively suppressing the kinetics of ion-ion reactions over broad m/z ranges. In the context of IIPT reactions, once a precursor has undergone a sufficient number of consecutive reactions to reduce its charge state such that it has an m/z within the desired m/z range, PIP dramatically slows the rate of additional reaction. If the reaction time is sufficiently extended, most of the product ions are “parked” at the highest product ion charge state that falls within the PIP m/z range.

Ion motion in radio frequency (RF)-only quadrupole field ion trapping devices is such that, for a given combination of RF field frequency and intensity, stably trapped ions have characteristic frequencies of motion that are wholly a function of m/z [17]. These characteristic frequencies increase or decrease as the trapping field intensity (applied trapping RF voltages) is correspondingly varied. Two-dimensional RF quadrupole linear ion trap (QLT) devices utilize auxiliary (approximately dipolar) AC fields which are superposed on top of the quadrupolar radial trapping RF field by application of differential AC voltages (single frequency or multi-frequency/broadband waveforms) between opposing quadrupole rod electrodes. Resonant kinetic excitation of ions of a particular m/z will occur when a frequency component of such an auxiliary AC waveform field matches (or is sufficiently close to) the corresponding characteristic frequency of the ions. Resonantly excited ions experience greater oscillatory motion (maximum displacements, maximum velocity, and average kinetic energy). Judicious construction (amplitude and frequency composition) and application of such auxiliary AC fields in concert with control of the RF trapping field intensity enable m/z-selective ejection of ions to an ion detector for m/z analysis, m/z isolation, and m/z-selective (narrow or broadband) kinetic activation to effect collision-induced dissociation (CID) [18].

The ion parking methods developed by the McLuckey group involve application of such auxiliary AC fields with intensities insufficient to induce dissociation or ejection of trapped ions. Instead, they serve to provide kinetic activation of product ions within selected m/z range(s) where further ion-ion reactions are not desired. The rate constants for gas-phase ion-ion reactions have a strong inverse dependence on the magnitude of the differential velocities of the reacting species. Stephenson and McLuckey derived an expression, given by Eq. (1), for the rate constant, *k*, where *v* is the magnitude of the differential velocity between the analyte precursor cations and the reagent anions, *z*_*1*_ and *z*_*2*_ are the integer charge states of the respective cation and anion reactants, *e* is the elementary charge, and *μ* is the reduced mass for the two reactant species [13].

1 $$ k= v\pi {\left(\frac{z_1{z}_2{e}^2}{\mu {v}^2}\right)}^2 $$

Equation (1) provides insight into important dependencies without having to resort to major numerical simulations. Reaction rates are proportional to the product squares of the anion and cation charge states. Since the IIPT reactions discussed herein involve singly charged reagent anions, the rate constant for each successive step of charge reduction depends on the square of the cation charge state. Reaction rate constants are inversely proportional to the cube of the magnitude of the differential velocity of the reacting ion clouds. Collective kinetic activation of product ions provided by an auxiliary AC field need only impart a modest increase in the velocity of the product ions to dramatically reduce the rate of subsequent charge reduction reactions. The ability to selectively arrest the kinetics of charge reduction of specific product ion m/z ranges during ion-ion reactions is advantageous for IIPT reactions as it enables concentration of charge-reduced analyte ions within a desired m/z range into one primary charge state.

Here, we describe modifications to a front-end ETD enabled hybrid dual cell QLT–Orbitrap mass spectrometer to enable PIP during IIPT reactions. We illustrate the increase in observed S:N achieved via concentration of multiple precursor charge states into a single charge state by use of IIPT and PIP. Further, we describe an improved ion parking method involving m/z-selective kinetic activation of both charge-reduced analyte protein product ions and IIPT reagent anions to more fully arrest ion-ion reactions within the parking m/z window. This enables more complete concentration of the highly charged protein IIPT analyte product ions into a dominant charge state. Finally, we describe an automated, data-dependent method for identification of proteins in a complex mixture by use of IIPT, PIP, and higher-energy collisional dissociation (HCD).

## Methods

### Sample Preparation and Chromatography

Ubiquitin (bovine) and apomyoglobin (horse heart) were purchased from Sigma-Aldrich (St. Louis, MO). One nanomole of lyophilized ubiquitin or apomyoglobin was reconstituted in a solution containing 40% acetonitrile and 0.1% acetic acid in water (all percentages expressed as *v*/*v*) to a final concentration of 2.5 picomoles/μL. The samples were directly infused at 60–100 nL/min via a laser-pulled (Sutter P-2000, Novato, CA) fused silica capillary (360 μm O.D. × 50 μm I.D.) emitter tip (~ 1 μm) and ionized by nano-electrospray ionization. The ESI source voltage was biased at 2.2 kV, and the heated capillary temperature was 250°C.

70S Ribosomes from *Escherichia coli* (B Strain) were purchased from New England BioLabs (Ipswich, MA). These were reconstituted in 100 mM ammonium bicarbonate, reduced and alkylated with 20× molar excess dithiothreitol and 40× molar excess iodoacetamide, respectively. For LC-MS/MS analysis, the protein mixture (3 μg total protein) was pressure-loaded onto a fused silica capillary precolumn (360 μm O.D. × 150 μm I.D.) packed 14 cm with Poroshell 300SB-C18 resin (5 μm diameter; Agilent Technologies, Santa Clara, CA). The precolumn was washed with ~ 20 column volumes of solvent A (solvent A: 0.3% formic acid in water) and connected to an analytical column (360 μm O.D. × 75 μm I.D.) equipped with an integrated, laser-pulled emitter tip (~ 1 μm), and packed 22 cm with the same resin. Proteins were eluted at 100 nL/min by use of the following gradient: 0–30% solvent B in 10 min, 30–70% B in 40 min, and 70–100% B in 10 min (solvent B: 0.3% formic acid, 72% acetonitrile, 18% isopropanol, 9.7% water). The eluted proteins were directly ionized by microelectrospray into the inlet of the mass spectrometer, described below.

### Instrument and Software Modification

We have previously reported modification of an Orbitrap Velos Pro™ (Thermo Fisher Scientific; Waltham, MA) to have the atmospheric pressure ionization inlet with ETD reagent ion source from an Orbitrap Fusion™ mass spectrometer to introduce reagent ions for ETD and IIPT (sulfurhexafluoride reagent) from the front of the instrument [19]. This enables C-trap accumulation of product ions from multiple cycles of ion accumulation, m/z isolation, and activation in the high pressure cell of the QLT prior to their injection into the Fourier transform (FT) Orbitrap analyzer. Such “multiple fill” methods have been demonstrated to improve S:N of MS/MS spectra [19, 20]. The instrument has since been upgraded with a compact, high-field Orbitrap [21, 22] (to make it a modified Orbitrap Elite™), and the reagent delivery system has been further modified to introduce perfluorormethyldecalin, PFMD (Technical Grade, Oakwood Chemical, West Columbia, SC) anions, an IIPT reagent, to the reagent ionization source. Briefly, 20 μL of PFMD was placed inside a stainless steel 1/8″ Swagelok™ cap, sealed to a Swagelok union connected to a 2-m-long fused silica capillary restrictor (360 μm O.D. × 10 μm I.D.). This restrictor was connected to the gas line providing the primary flow of nitrogen to the discharge source via a second 10 cm, 25 μm I.D. PEEKsil™ restrictor. The Swagelok cap served as the PFMD reservoir and was maintained at room temperature. The fused silica restrictor was the primary limiter of the flow of PFMD vapor to the reagent source while the PEEKsil restrictor allowed the reservoir to be refilled and the fused silica restrictor to be replaced without venting the instrument.

The instrument control software was modified to calculate the AC voltage waveforms for PIP with the same functions used for calculation of m/z isolation waveforms. The resultant array of voltages was stored in the instrument computer’s waveform memory for playback at the appropriate time. The waveforms were composed of frequency components corresponding to the m/z range defined in the experiment and spaced every 500 Hz (waveform periods were 2 ms). In the frequency domain, these AC voltage waveforms may be conceived of as frequency combs with the tine lengths corresponding to the amplitudes of the individual frequency components. These PIP AC voltage waveforms were applied continuously to all three segments of the high pressure QLT x-rod pairs for the duration of the user-defined IIPT reaction intervals. A Tektronix MDO3014 (Tektronix Co. Beaverton, OR) digitizing oscilloscope with FT functionality was used to verify the frequency composition of PIP waveforms by recording and Fourier transforming the waveform signal at the input and output for the waveform drive amplifier in the instrument electronics. An example PIP AC waveform spectrum (magnitude mode) is shown in Figure 3. The waveform applied during IIPT included frequencies corresponding to the reagent (PFMD, m/z 512) ± 10 Th at a normalized activation amplitude (instrument arbitrary units) of 0.02, as well as frequencies corresponding to a 1000 m/z range within which the IIPT product ions were to be “parked” at a normalized activation amplitude of 0.09. The m/z range for IIPT product ion parking was varied based upon experimental/sample conditions and was adjusted as detailed below.

### Mass Spectrometry

IIPT/PIP MS/MS spectra were acquired under the following conditions and instrument settings: 120,000 resolving power at m/z 400; 300–4000 m/z scan range; 1E5 precursor AGC target; 3E5 reagent (PFMD) AGC target; 1 microscan/spectrum; 5 Th precursor isolation window or 400 Th precursor isolation window centered at m/z 800 (for the ribosomal protein mixture); 10–150 ms IIPT reaction time (100 ms for the ribosomal protein mixture); 10 product ion fills of the C-trap (1E6 cumulative AGC target). The PIP AC voltage waveforms were applied only during the IIPT reaction segments (scan function) of the experiment. The waveform included all frequencies corresponding to a 1000 Th range starting at either m/z 1450 (apomyoglobin only) or m/z 1150 (protein mixtures) at a normalized activation amplitude of 0.09. Where specified, the reagent activation window included all frequencies corresponding to a 20 Th window centered on m/z 512 at a normalized activation amplitude of 0.020.

For online, data-dependent analysis of *E. coli* ribosomal proteins, MS1 scans were acquired under the following conditions: 120,000 resolving power at m/z 400; 300–2000 m/z scan range; 1E6 Full MS AGC target, 1 microscan/spectrum. This was followed by acquisition of an “m/z isolation-only” MS/MS spectrum taken with the same resolving power, scan range, isolation width, and cumulative AGC target as the subsequent IIPT/PIP MS/MS spectrum (described above). Following IIPT/PIP, a data-dependent (most abundant from PIP/IIPT MS/MS) HCD MS^3^ spectrum was acquired under the following conditions: 120,000 resolving power at m/z 400; 200–2000 m/z scan range; 1E5 precursor AGC target; 1 microscan/spectrum; 5 Th isolation window; 35% normalized collision energy; 10 product ion fills of the C-trap (1E6 cumulative AGC). API source CID “energy” was set to 35 V across all scan types. Data-dependent exclusion utilized a repeat count of 3, 60-s repeat duration, and 120-s exclusion duration. Charge states excluded from MS^N^ selection included + 1 and + 2.

### Data Analysis

Data derived from the *E. coli* ribosomal protein mixture were searched using ProSight PD™ (Thermo Fisher Scientific PD version 2.1, ProSightPC version 4.0) against an *E. coli* database (UniProt Swiss-Prot 03/26/2016). The Spectrum Selector node was set to search HCD (MS3) spectra with the precursor parameter set to "Use MS(n-1) Precursor", allowing intact mass to be derived from the previous MS2 spectrum. A three-step search was then performed on the HCD spectra. The first step was an absolute mass search with a 100-Da precursor mass tolerance, 10-ppm fragment mass tolerance, and delta m mode off. Spectra that did not score with high confidence were then passed to a biomarker search with a 10-ppm precursor mass tolerance and a 10-ppm fragment mass tolerance. Finally, spectra that were still not confidently assigned were passed to a second absolute mass search with a much wider 1000-Da precursor mass tolerance, 10-ppm fragment mass tolerance, and delta m mode on. All search modes utilized the XTract algorithm (Thermo Fisher Scientific) with a precursor S:N threshold of 3 and a fragment ion S:N threshold of 1.5. Static modification of carbamidomethyl cysteine was included. All protein identifications returned by the three-step search were manually validated by visual inspection of the data using Xcalibur™ Qual Browser software (Thermo Fisher Scientific version 4.0). Theoretical intact masses and fragment ion m/z were calculated using an in-house-developed mass calculator. Interpretation of HCD MS^3^ data was performed on the unprocessed raw spectra. Interpretation of intact mass data derived from IIPT/PIP MS^2^ spectra was performed following deconvolution with the Qual Browser-embedded Xtract algorithm operated with default parameters and a S:N threshold of 5.

## Results

**Figure 1:**
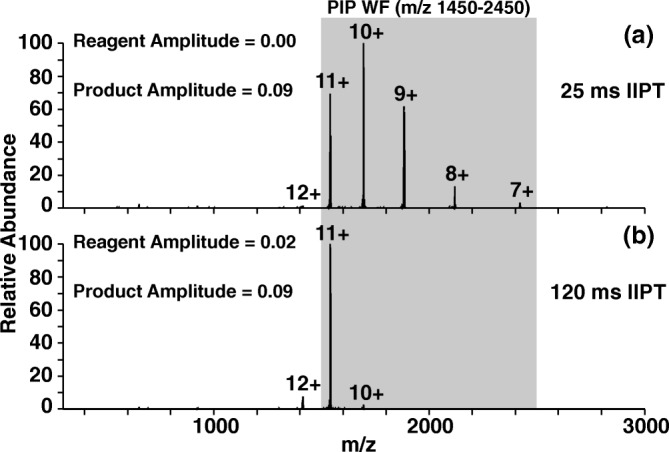
(**a**) MS/MS spectrum of [M+26H]^26+^ ions of apomyoglobin (m/z 653) following 25 ms of IIPT with PFMD reagent anions. During the reactions, a waveform including frequency components corresponding to m/z 1450–2450 was applied to x-rods of the QLT at a normalized activation amplitude of 0.09. (**b**) MS/MS spectrum of [M+26H]^26+^ ions of apomyoglobin (m/z 653) following 120 ms of IIPT with PFMD reagent anions. During the reactions, a waveform including frequency components corresponding to the reagent (m/z 512) and m/z 1450–2450 (gray) was applied to x-rods of the high-pressure QLT at a normalized activation amplitude of 0.02 and 0.09 for the reagent and product ion parking window, respectively

In our early attempts to implement parallel ion parking on our QLT-Orbitrap instrument, we constructed the PIP waveforms in accordance with the teachings of the McLuckey group [15, 16], where the intensity of each waveform frequency component corresponding to the m/z range for product ion parking was of uniform amplitude and all other frequencies had zero amplitude. With our instrument, we found that such PIP AC waveforms were not highly effective at selectively suppressing further ion-ion reactions for the relatively high charge state product ions derived from intact proteins. Attempts to increase the amplitude of the PIP waveform and thereby increase product ion kinetic excitation within the PIP m/z range resulted in collision-induced fragmentation of the charge-reduced protein product ions rather than improved suppression of further charge reduction. Shown in Figure 1a is an IIPT MS/MS spectrum resulting from the [M+26H]^26+^ (m/z 653) charge state of apomyoglobin following a 25-ms reaction with PFMD radical anions and is representative of our initial results with PIP waveforms modeled on those described by the McLuckey group in their original work. During the reaction, an auxiliary AC waveform composed of only a uniform intensity comb of all frequencies corresponding to the m/z range 1450–2450 was applied between the QLT x-rod electrodes. The [M+11H]^11+^ charge state isotopic peak cluster has an m/z of 1542 and corresponds to the first sequentially charge-reduced product ion species to fall within the intended m/z range for product parking. Ideally, all of the charge-reduced product ion signal should be concentrated in this charge state; however, we observed that the majority (more than 2/3) of the product ion signal resided in the isotopic peak clusters corresponding to products of further consecutive IIPT reactions that occurred despite the application of the PIP waveform. The large difference in the intensity of the [M+12H]^12+^ species relative to that of the [M+11H]^11+^ charge-reduced form indicates that the parking AC waveform voltage greatly slowed the rate of reaction for the charge states within the parking m/z window—the 12+ charge state was almost completely depleted. However, the reduction in IIPT reaction rate was not sufficient so as to suppress further consecutive charge reduction reactions as isotopic peak clusters from the 10+, 9+, and 8+ charge states were observed in high relative abundance.

When we first attempted to implement PIP, we modified the instrument code to generate PIP AC waveforms with no means to verify that our code generated waveforms with the frequency composition that we intended. We used these AC waveforms and were unable to obtain full suppression of ion-ion reactions within the intended parking widows. Concerned that perhaps the problem was that we were not producing PIP waveforms with the intended frequency composition, we obtained and connected a digital oscilloscope with discrete Fourier transform capabilities to the instrument’s AC waveform drive amplifier to check that the frequency composition of the PIP waveforms we were creating were as we intended. We discovered that our initial waveform construction produced low but non-zero tine amplitudes throughout the range of frequencies corresponding to most of the stable range of m/z values in the QLT. Therefore, precursor ions, product ions outside of the parking band, and reagent ions were also subjected to some degree of kinetic activation. We remedied these mistakes in our PIP AC waveform construction instrument code and were able to generate AC waveforms with the desired frequency composition (uniform non-zero tine amplitudes within the frequency band corresponding to the m/z range for product ion parking and zero amplitude tines for all other frequencies). These “proper” PIP AC waveforms produced the incomplete parking shown in Figure 1a (as described above), which was actually a less complete degree of ion parking than the best results we had observed with the inadvertently “incorrectly” constructed waveforms we initially utilized. This prompted much further experimentation and led to the conclusion that the inadvertent kinetic activation of the reagent ions was what produced the superior parking efficacy with the improperly constructed PIP waveforms.

The effect of mild kinetic activation of the reagent anions on IIPT reaction rates with highly charged precursor ions and charge-reduced product ions is demonstrated in Supplemental Figure 2. Panel a shows the experimentally obtained product ion spectrum from IIPT of the 26+ charge state of apomyoglobin following a 10-ms reaction time without any kind of PIP AC waveform applied (no m/z-selective product ion kinetic activation and no m/z-selective reagent kinetic activation). The most abundant charge-reduced product ion species is the 11+ charge state. Panel b shows the identical experiment but with an AC waveform constructed to provide m/z-selective kinetic activation only to the reagent anions applied during the IIPT reaction. Here, the most abundant product ion species is the 19+ charge state, and it is clear that the reaction rates for all successive generations of IIPT reactions were considerably reduced.

**Figure 2:**
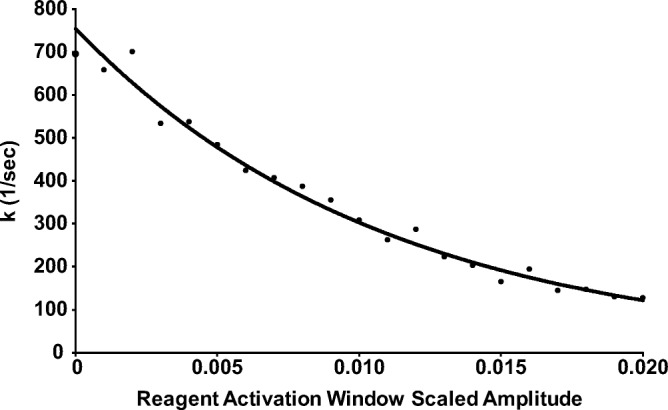
Plot of experimentally determined IIPT reaction rate constant, k, of [M+13H]^13+^ ions of ubiquitin (m/z 660) with PFMD reagent anions as a function of the scaled amplitude of the reagent activation waveform frequency components

When suitably large populations of reagent anions are used relative to precursor ion populations, the reactions in RF quadrupole ion traps behave, in good approximation, according to pseudo-first-order kinetic theory. The abundance of unreacted precursor ions will decay exponentially with reaction time [13, 23, 24]. Reaction rate constants are derived by exponential fit to the post-reaction abundance of the m/z-selected precursor ion versus ion-ion reaction period. An example of the effect of reagent activation on reaction rate constant (k) is illustrated in Figure 2. The experimentally determined rate constant of IIPT reactions of the [M+13H]^13+^ charge state of ubiquitin (m/z 664) with PFMD reagent anions is shown as a function of the amplitude of the uniform amplitude band of AC waveform component frequencies applied to produce m/z-selective kinetic excitation of the reagent ions. In this series of experiments, the AC waveform was composed of only those frequency components appropriate for reagent kinetic activation. Here, the component frequency amplitudes of the AC waveform are expressed in the normalized units used in calculating the waveform and are proportional to the actual amplitude of the waveform voltage frequency components applied to the opposing electrodes of the linear trap. During the IIPT reaction period, the RF trapping field voltages were such that the Mathieu q for the PFMD reagent was 0.55 (the QLT trapping field frequency was 1.150 MHz). The rate constant, k, for each level of reagent excitation was determined by fitting an exponential decay function to the abundance of the remaining precursor ion, ubiquitin 13+, for a series of MS/MS IIPT spectra where the IIPT reaction times were progressively extended. Initial precursor and reagent ion populations were the same for all experiments. The data in Figure 2 demonstrate that kinetic activation of the reagent ions progressively reduces the reaction rate constant for the reagent-analyte (protein) precursor ion-ion reaction from its initial value to about 1/7th its initial value. The ion-ion reaction rate constants for all analyte cations (precursor and charge-reduced products) can be reduced in a controlled manner by increasing the degree of reagent kinetic activation.

An understanding of how kinetic activation of the reagent ions would result in the overall slowing of ion-ion reaction rates in the QLT led to a hypothesis regarding how the inadvertent kinetic activation provided by our “incorrectly” constructed PIP AC waveforms might improve the efficacy of ion parking for highly charged product ions. Since preceding generations of intermediate charge-reduced product ions that have m/z values outside the m/z range for ion parking would have near thermal kinetic energies (trapped ions in conventional RF QLT analyzers kinetically relax through collisions with helium atoms and have near thermal velocity distributions, and thus near thermal kinetic energies), we reasoned that the first generation of IIPT product ions that have m/z values within the m/z range for ion parking would initially also have the near thermal kinetic energies. The difference in mass between a protein cation and IIPT reagent anion would be such that, even if the reagent ions were kinetically excited well above thermal energies, the momentum transfer during the charge-transfer interaction would result in a minimal increase in kinetic energy in the charge-reduced product ion. Kinetically cooling collisions with the helium background gas would ensure that there would be no buildup of kinetic energy through consecutive reactions. The rate of kinetic energy uptake of a newly created charge-reduced product ion within the parking m/z range would be relatively slow as it would be counter-balanced by kinetic energy dissipation through collisions with helium. The IIPT reaction rates of charge-reduced products of protein precursors are still very high due to the still relatively high charge states they exhibit (*k* ∝ *z*^2^). We hypothesized that the initially observed, undesired charge-reduced product ions within the parking m/z range (Figure 1a 10+ through 7+) are the result of continued exposure to reagent anions at low differential velocities during the interval of time (hundreds of μs to 2 ms) between when a product ion is created within the parking m/z range and when it achieves a sufficient level of kinetic activation to effectively shield it from further IIPT reactions. When the reagent ions are kinetically excited, the likelihood of further IIPT reactions for newly formed product ions within the parking m/z window during the period of “vulnerability” is reduced.

**Figure 3:**
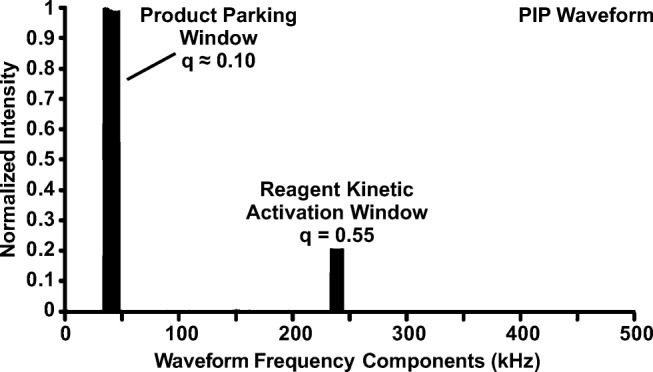
Example frequency composition of a broadband PIP waveform with mild reagent kinetic activation. The product ion parking window includes frequency components corresponding to m/z 2400–3400. The Mathieu *q* of the reagent (m/z 512) was set to 0.55

With this in mind, we began deliberately adding a band of component frequencies to our PIP waveforms that would provide reagent kinetic excitation in addition to kinetic excitation of product ions. An example of the frequency composition of a parking waveform with frequency components for reagent ion kinetic activation is shown in Figure 3—in addition to the comb of frequencies corresponding to the product ion parking window, we applied a low-amplitude secondary comb of frequencies to provide kinetic excitation to the reagent ions (PFMD radical anion, m/z 512 ± 10 Th) in order to slow the overall kinetics of the IIPT reactions. These “improved” PIP AC waveforms provided demonstrably more thorough suppression of ion-ion reactions for product ions within the parking m/z range, allowing efficient concentration of the vast majority of the product ion signal into a single charge state (Figure 1b). Approximately 85% of the total ion signal is parked in the 11+ charge state. The 10+ charge state has a relative abundance that is less than 5%, indicating that further IIPT reactions of the 11+ charge state were substantially arrested. Note that since the reagent kinetic activation slowed the reaction rates for all analyte ions, the reaction time was extended from 25 to 120 ms to achieve this result.

**Figure 4:**
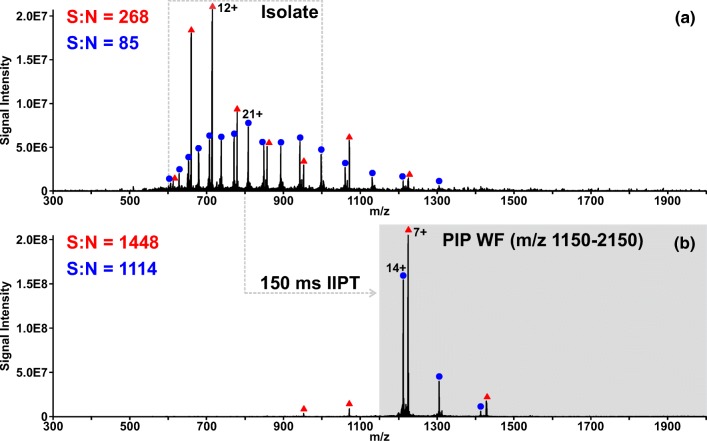
(**a**) Positive ESI MS^1^ spectrum depicting charge state distributions for intact ubiquitin (▲) and apomyoglobin (●). (**b**) MS/MS spectrum following isolation of all precursors between m/z 600 and 1000 (from **a**) and 150 ms IIPT. A PIP waveform containing frequency components corresponding to m/z 1150–2150 (gray) and mild kinetic activation of the reagent was applied to the x-rods of the high-pressure QLT for the duration of the IIPT reaction. The S:N (red = ubiquitin; blue = apomyoglobin) of the most abundant charge states of each protein are indicated in both spectra

In order to be effective at performing IIPT/PIP on mixtures of unknown proteins, it is necessary to use wide isolation windows to ensure that the majority of charge states of all coeluting proteins are captured, and the total ion current is maximized in the resulting IIPT/PIP product ion spectrum. For the experiment shown in Figure 4, a 1:1 mixture of ubiquitin (8.6 kDa) and apomyoglobin (16.9 kDa) was directly infused at a concentration of 2.5 μM. Panel a depicts the positive ESI mass spectrum. Note that while both analytes were present at approximately equivalent concentrations in the MS1 spectrum (Figure 4a), the most abundant charge state (21+) of the larger protein (apomyoglobin) has a signal intensity that is approximately 30% relative to the most intense charge state of ubiquitin (12+). This MS1 spectrum exhibits isotopic m/z peak clusters corresponding to 16 charge states of apomyoglobin and 8 charge states of ubiquitin. In the subsequent MS2 spectrum, a 400-Th window (600–1000 m/z) was isolated and then charge reduced by IIPT. A PIP waveform including frequency components corresponding to the product ion parking window (m/z 1150–2150) and mild kinetic activation of the IIPT reagent anions was applied to the QLT x-rods during the reaction. The resulting MS/MS spectrum is depicted in Figure 4b. In this case, it was necessary to use a relatively long IIPT reaction time (150 ms) to effectively charge reduce the ubiquitin precursor ions so that the majority of the products were observed in the parking window. As a consequence, apomyoglobin cations underwent further charge reduction after being reduced to the 14+ charge state. Despite this, the bulk of the total ion current for both proteins is “parked” in just 2–3 charge states, and more than 2/3 of the signal for each protein is concentrated in the most abundant charge state. Further, the S:N for the m/z peaks for the most abundant charge states of each protein was improved by a factor of 5 for ubiquitin and by a factor of 13 for apomyoglobin relative to the S:N of m/z peaks corresponding to the most abundant charge states in the MS1 spectra.

**Figure 5:**
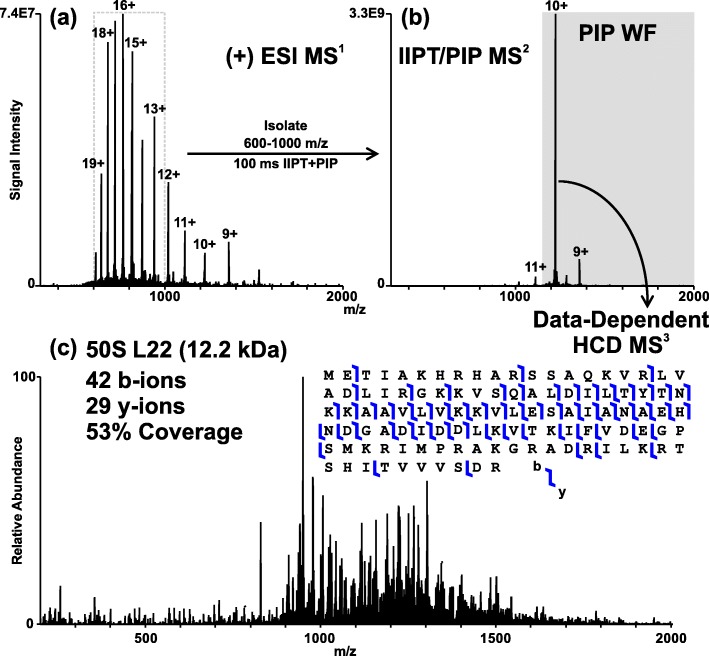
Scheme for HPLC-MS/MS analysis of *E. coli* ribosomal proteins. (**a**) Positive ESI MS^1^ spectrum taken from 300 to 2000 m/z. (**b**) All precursors between m/z 600 and 1000 were isolated and subjected to 100 ms IIPT with PIP performed on product ions falling between m/z 1150 and 2150 (gray). (**c**) Precursors are data-dependently selected from the IIPT/PIP MS^2^ for sequence analysis by HCD fragmentation in the subsequent MS^3^

To demonstrate the utility of PIP during IIPT, we applied the technology to the analysis of intact proteins that constitute the *E. coli* ribosome. We chose this model because it is sufficiently complex (54 proteins), and the proteins comprise a wide range of molecular weights (3.5–61 kDa), with most falling somewhere between 10 and 30 kDa. Disulfide bonds were reduced and alkylated with iodoacetamide and approximately 3.33 μg total protein (corresponding to roughly 2 pmol/protein) was loaded onto a reversed-phase HPLC column and analyzed by online LC-MS/MS. Figure 5 illustrates the sequence of FTMS scans that were repeatedly acquired during the analysis. First, a positive ESI full MS spectrum was taken (5a). In the second MS spectrum, a precursor m/z window of 600–1000 Th was isolated, and the precursors subjected to 100 ms IIPT with PFMD reagent anions. During the reaction, a PIP waveform was applied which provided mild kinetic activation of the reagent and a broadband PIP window targeting m/z 1150–2150. Broadband parking waveforms are necessary to park multiple protein charge-reduced products in the absence of a priori knowledge of the precursor mass (m/z and z) or product ion m/z. As long as the m/z of one charge-reduced product ion from each precursor species (limited to 600–1000 m/z) fell somewhere between m/z 1150 and 2150, we could expect to observe a corresponding isotopic peak cluster in the resulting IIPT/PIP MS^2^ spectrum (5b). Parked, charge-reduced precursors (intact IIPT products) were then data-dependently selected for analysis by MS3. In the final MS experiment of the cycle, the data-dependently selected IIPT product ion was isolated and fragmented by HCD (5c). For all FTMS scan events, the cumulative AGC target was 1E6 charges. The average cycle time during the period of protein elution was approximately 7.5 s.

**Figure 6:**
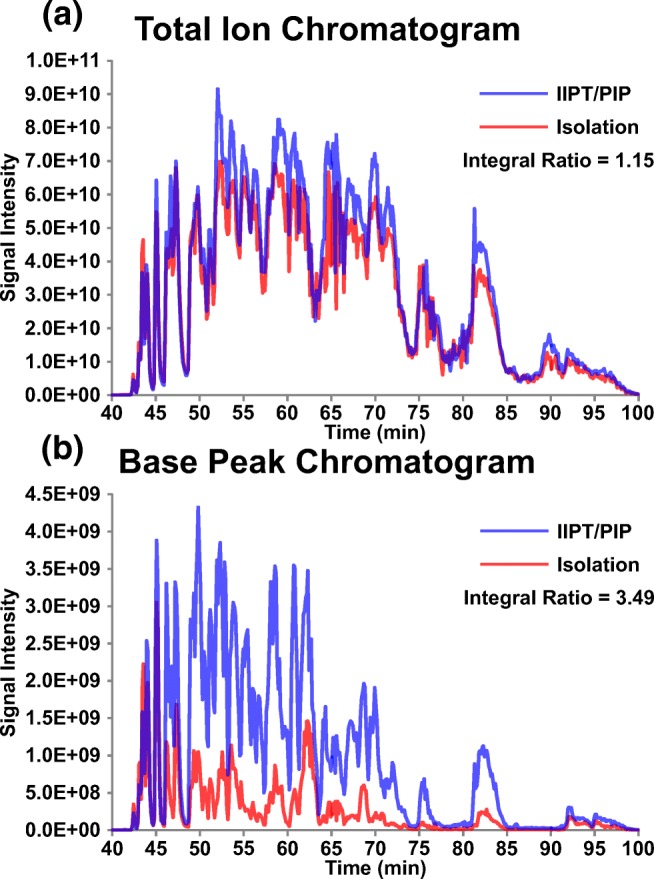
(**a**) Total ion and (**b**) base peak chromatograms obtained by HPLC-MS/MS analysis of *E. coli* ribosomal proteins. Traces in red are derived from the 400-Th isolation (m/z 600–1000) MS^2^ spectra (no IIPT/PIP), and traces in blue are derived from the subsequent IIPT/PIP MS^2^. Both scan types utilized equivalent cumulative ion targets. The ratio of the chromatogram area for each scan type is indicated (integral ratio)

Figure 6a shows traces of total ion chromatograms during the period of protein elution (60-min gradient). The chromatogram derived from the 400-Th isolation is depicted in red and the chromatogram derived from the subsequent IIPT/PIP MS^2^ spectra is in blue. The ratio of the peak areas for each chromatogram was calculated in Xcalibur™ and should theoretically be 1 and experimentally show reasonably good agreement with a ratio of 1.15 (IIPT/PIP:isolation). Here, the blue trace is consistently slightly higher in intensity than the red trace. We suspect that this is due to a decrease in collisional cross-section (less denatured gas-phase conformation) leading to a slower decay in the corresponding Orbitrap image current transients for the IIPT reaction product due to their reduced charge. Figure 6b shows the base peak chromatograms for each scan type. Here, improvements in signal intensity of the most abundant species are readily apparent. The integral ratio between the two chromatograms is approximately 3.5, though the magnitude of improvement is highly dependent upon the charge states and ion-ion reaction rates of the proteins eluting at any given time.

**Figure 7:**
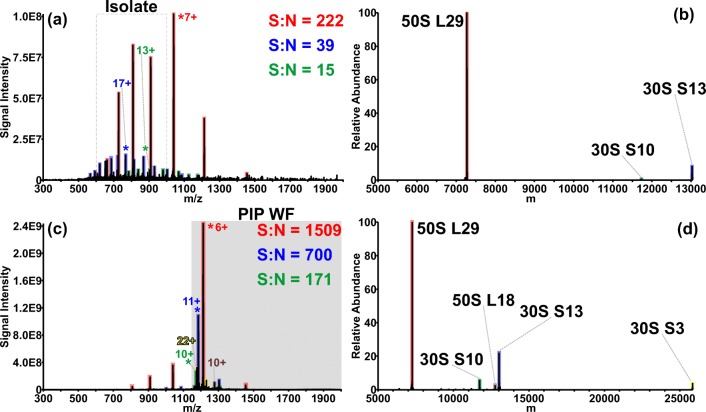
(**a**) Positive ESI MS^1^ depicting charge state distributions of three distinct, co-eluting proteins (50S L29/red, 30S S13/blue, and 30S S10/green). Precursors between m/z 600 and 1000 were isolated and subjected to IIPT/PIP. (**b**) Spectrum obtained following Xtract deconvolution of (**a**) with signals corresponding to three proteins. (**c**) Subsequent IIPT/PIP MS^2^ spectrum. Signals derived from two additional proteins (30S S3/yellow, 50S L18/brown) are indicated. (**d**) Spectrum obtained following Xtract deconvolution of (**c**) with signals corresponding to five proteins. S:N of the most abundant charge states of the three most abundant proteins are indicated in (**a**) and (**c**)

Traditionally, intact mass measurement data is obtained from positive ESI MS^1^ spectra. In addition to the gain in S:N for a given analyte following IIPT/PIP, we subsequently observed improvements in the data analysis process, specifically an increase in the number of species observed following deconvolution of IIPT/PIP MS^2^ spectra compared to the MS^1^ spectra. An example is shown in Figure 7. Manual examination of the MS1 spectrum shown in 7a (retention time 61.79 min) revealed charge state distributions derived from three distinct proteins (indicated in red, blue, and green). The signal to noise ratios of the most abundant charge states (*) for each protein are given in the upper right hand corner of the panel and ranged from 15 to 222. These three proteins are observed in the m/z-to-mass deconvolved spectrum (Xtract™) shown in 7b and range in size from 7.2 to 13 kDa. Following 400-Th isolation and IIPT/PIP, signals derived from five distinct proteins are readily apparent (7c; retention time 61.81 min). S:N of the most abundant charge states of each of the three initially observed proteins are improved by a factor of 6.8 (50S L29) to 17.9 (30S S13). All five species appear in the m/z-to-mass deconvolved spectrum (7d; 7.2–25.9 kDa) and were selected for HCD MS^3^ which, combined with intact mass measurement, enabled assignment of their identities. This is a modest example of the improvement in the overall number of species observed following deconvolution of IIPT/PIP MS^2^ spectra. Another example is shown in Supplemental Figure 3 where we observed that the number of deconvolved spectral peaks with masses (greater than 5000 Da) more than double, from 10 derived from 400-Th isolation spectrum, to over 20 following IIPT/PIP. However, not all of these potential MS3 precursor species were selected for MS/MS due to the low duty-cycle times inherent to FTMS analysis.

Note that the relative abundances of each protein reflected in the m/z-to-mass deconvolved spectra shown here are not accurate representations of the abundance of each protein. Due to the differences in charge state distributions among the coeluting proteins, more charge states of some of the proteins are captured in the 400-Th isolation than other proteins. In this example (Figure 7), the vast majority of charge states derived from 30S S13 are isolated, but charge states derived from 50S L29 are under sampled as they fall outside the 400-Th isolation window. Additionally, the precursor ion charge states of the 50S L29 protein are considerably lower (owing to the small size of the protein). Thus, not all the precursor ions derived from this protein are charge reduced to the PIP window. This suggests that in order to obtain quantitative information, isolation windows should be widened and IIPT reaction times extended to ensure that all IIPT product ions fall within the parking window. For the qualitative purposes of this experiment, the parameters utilized were sufficient to demonstrate the performance of the methods described here.

The data obtained in this single LC-MS/MS experiment were searched using ProSight PD™ against an *E. coli* database. The spectrum selector node was set to search HCD MS^3^ spectra with the precursor parameter set to “Use MS(n-1) Precursor”, which allowed intact mass data to be derived from the previous IIPT/PIP MS^2^ spectrum. The search returned the identities of 46 of the 54 ribosomal proteins (exported search results can be found in Supplemental Table 1). Three additional, non-ribosomal proteins were also identified, each closely related to ribosomal function. Each assigned protein was manually verified and no false positive results were obtained from the search. In addition to the 46 ribosomal proteins identified in the search, 6 more were manually identified. The remaining two ribosomal proteins were observed by intact mass, but not selected for HCD fragmentation. One of these was 30S S1 (61 kDa) which was observed in experiments in which the ion trap was scanned to generate the IIPT/PIP MS2 spectra (Supplemental Figure 4) but was never discernable in corresponding experiments where the Orbitrap analyzer was used to generate the MS2 FTMS spectra.

## Conclusions

Using a suitably modified Orbitrap Elite mass spectrometer, we have demonstrated the utility of an LC-MS-based method for analysis of a moderately complex mixture of intact proteins that utilizes ion-ion proton transfer (IIPT) for charge reduction coupled with parallel ion parking (PIP). IIPT and PIP were used to concentrate a multiplicity of charge states to which an eluting protein was initially ionized into a lower primary charge state. The broad product ion m/z range for arresting IIPT reactions enabled by PIP allows the concentration of multiple protein species into respective primary charge states at the same time and avoids the necessity of prior knowledge of their intact mass. Data-dependent selection of the most abundant charge state of the most abundant protein in simplified IIPT-PIP MS^2^ spectra and subsequent HCD MS^3^ fragment ion spectra provided data that was amenable to a database search. The S:N of intact protein ion signals is significantly improved, enabling observation of protein species that are not observed in broadband MS1 spectra.

Further, to achieve the highly effective ion parking necessary to maximize concentration of each ionized protein species into a lower primary charge state through IIPT, we employed an improved PIP methodology. Our approach to PIP not only provides kinetic excitation of the product ion m/z range as originally described by McLuckey and co-workers, it also utilizes simultaneous kinetic activation of the reagent ions to slow the overall kinetics of IIPT and greatly reduce the probability of further ion-ion reactions. This improved method enables highly effective parking of protein ions with relatively high charge states and very high rates of reaction when they are not kinetically excited. While in this work, reagent activation has been applied to IIPT, we know that this approach is applicable to ETD to preserve highly charged product (sequence) ions from further dissociative ETD reactions. This application will be the subject of a follow-up manuscript (in preparation).

Mass spectrometric analysis of intact proteins in complex mixtures is fraught with challenges. Co-elution of multiple proteoforms and distribution of ion signal for each proteoform among multiple charge states—each having multiple isotopic m/z peaks—leads to the low S:N inherent to the currently utilized approaches for analysis of these large biological molecules. It is clear from the recent efforts of the Brodbelt and McLuckey laboratories [25, 26] that ion parking and parallel ion parking techniques are potentially advantageous in this challenging MS application area. We believe that the combination of IIPT and parallel ion parking with reagent activation (PIP-RA) will, with further improvements in the methodology and accompanying improvements to MS instrumentation, become a powerful tool in the study of intact protein mixtures. We are excited about what the future holds for intact protein mass spectrometry as applications of such technology have the potential to revolutionize our understanding of numerous biological systems.

## Electronic supplementary material

ESM 1 (PDF 654 kb)

ESM 2 (XLSX 385 kb)

## Abbreviations

ESI: electrospray ionization
IIPT: ion-ion proton transfer
PIP: parallel ion parking
RF: radio frequency
QLT: quadrupole linear ion trap
FTMS: Fourier-transform mass spectrometry
PFMD: perfluoromethyldecalin
HCD: higher-energy collision dissociation
CID: collision-induced dissociation
ETD: electron-transfer dissociation
S:N: signal-to-noise
PIP-RA: parallel ion parking with reagent activation
