# Supplementary material for: Ion-Ion Proton Transfer and Parallel Ion Parking for the Analysis of Mixtures of Intact Proteins on a Modified Orbitrap Mass Analyzer
Source: J Am Soc Mass Spectrom. 2019 Aug 7;30(10):2163–73. doi: 10.1007/s13361-019-02290-8 (PMC6805958; doi:10.1007/s13361-019-02290-8)
Supplement: Supplementary file 1 — (PDF 654 kb) [file 13361_2019_2290_MOESM1_ESM.pdf]

Supplemental Information

for

Ion-ion proton transfer and parallel ion parking for the analysis of mixtures of intact proteins on a modified orbitrap mass analyzer

Scott A. Ugrin[1], A. Michelle English[1], John E.P. Syka[2], Dina L. Bai[1], Lissa C. Anderson[3], Jeffrey Shabanowitz[1], Donald F. Hunt[1,4*]

1. Department of Chemistry, University of Virginia, Charlottesville, VA, 22904, USA
2. Thermo Fisher Scientific, San Jose, CA 95134, USA
3. Ion Cyclotron Resonance Program, National High Magnetic Field Laboratory, Tallahassee, FL, 32310, USA
4. Department of Pathology, University of Virginia, Charlottesville, VA, 22908, USA

* corresponding author, dfh@virginia.edu

Running Title: Parallel ion parking during proton transfer reactions

Supplemental Table 1 (submitted as a separate *.xls* file) contains exported ProSight PD™ search results of data derived from the *E. coli* ribosomal protein HPLC-MS/MS experiment. Search parameters are detailed in the methods section of the manuscript.

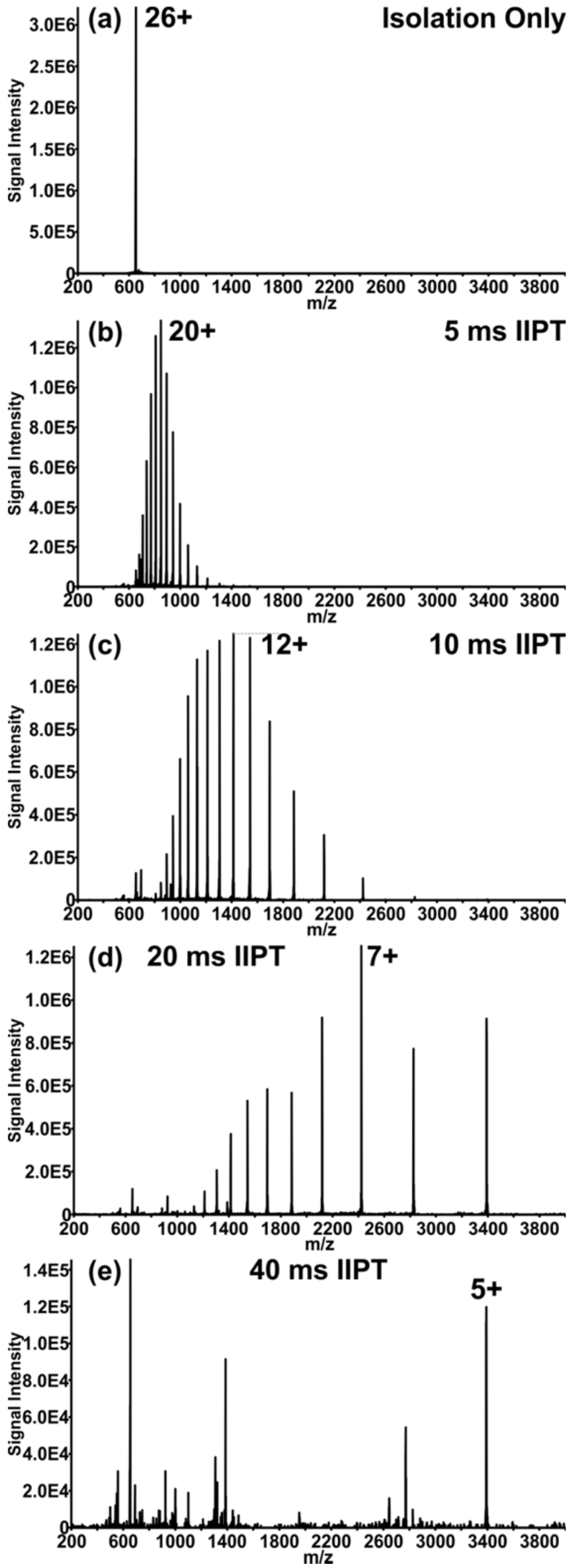

Supplemental Figure 1. (**a**) IIPT MS/MS spectrum depicting isolation of the $[M+26H]^{26+}$ charge state of intact apomyoglobin produced by positive ESI under denaturing conditions. (**b-e**) MS/MS spectra depicting charge reduction of the $[M+26H]^{26+}$ charge state of intact apomyoglobin following 5, 10, 20, and 40 ms of IIPT, respectively. The charge states of the most abundant product ions observed in the spectra following each reaction duration are indicated.

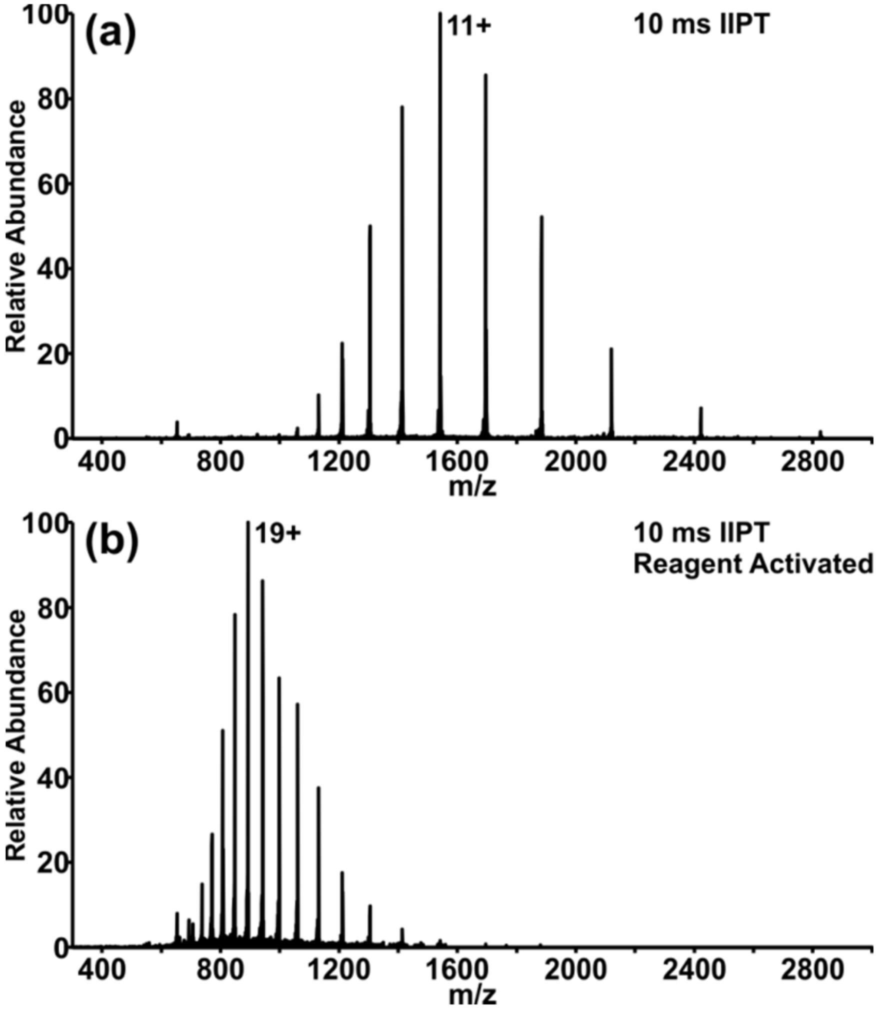

Supplemental Figure 2. MS/MS spectra depicting charge reduction of the $[M+26H]^{26+}$ charge state of intact apomyoglobin following 10 ms of IIPT with no reagent kinetic activation (**a**) and with mild reagent kinetic activation (**b**). The charge states of the most abundant product ions observed in the product ion spectra are indicated.

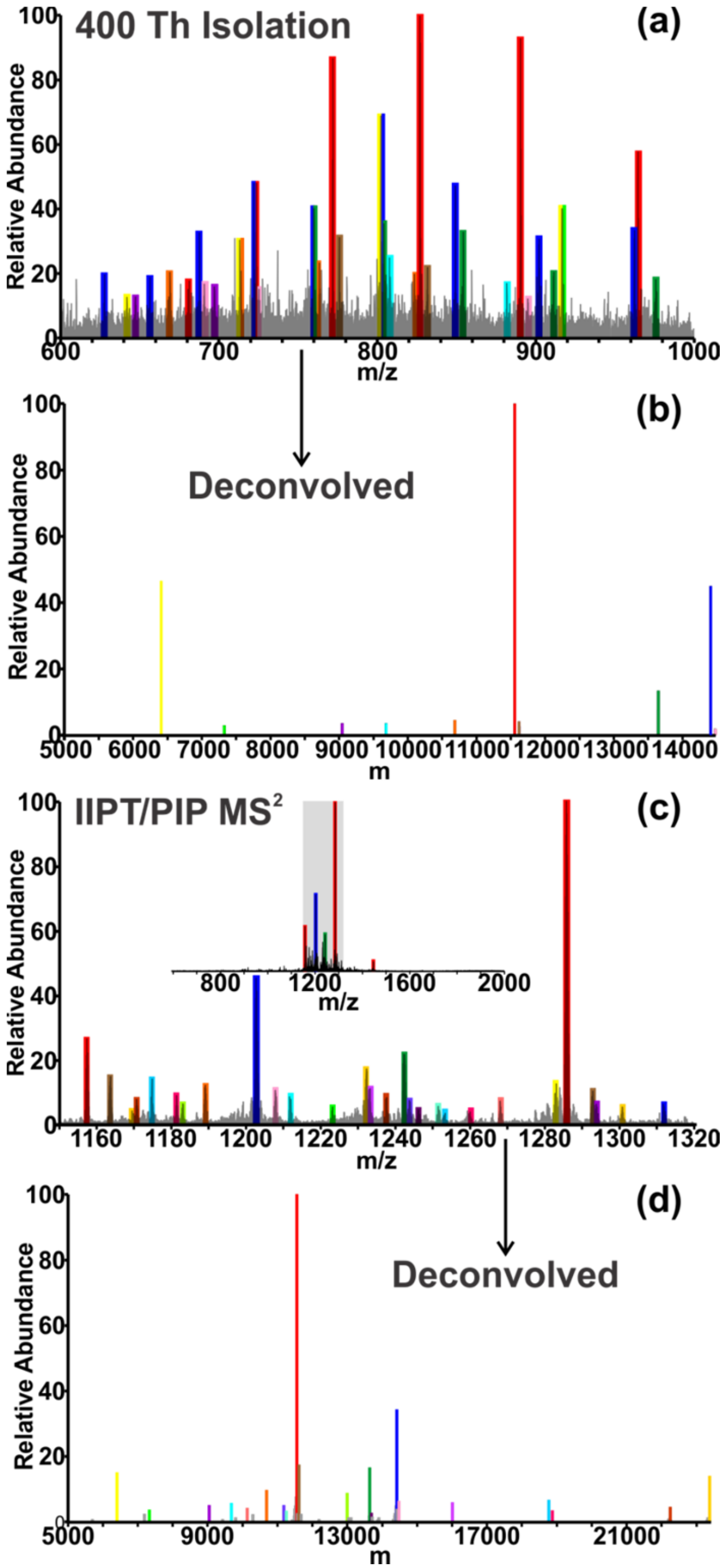

Supplemental Figure 3. (**a**) 400 Th isolation MS^2 spectrum of precursors derived from several co-eluting proteins. (**b**) Spectrum obtained following Xtract deconvolution of **a** with signals corresponding to 10 proteins. (**c**) Scale-expanded segment (grey region of inset) of the subsequent IIPT/PIP MS^2 spectrum. The inset shows an expanded view of the spectrum. (**d**) Spectrum obtained following Xtract deconvolution of **c** with signals corresponding to more than 20 proteins. Species of m < 5000 are not shown in the deconvolved spectra. Signals derived from the same protein are color coded across all spectra.

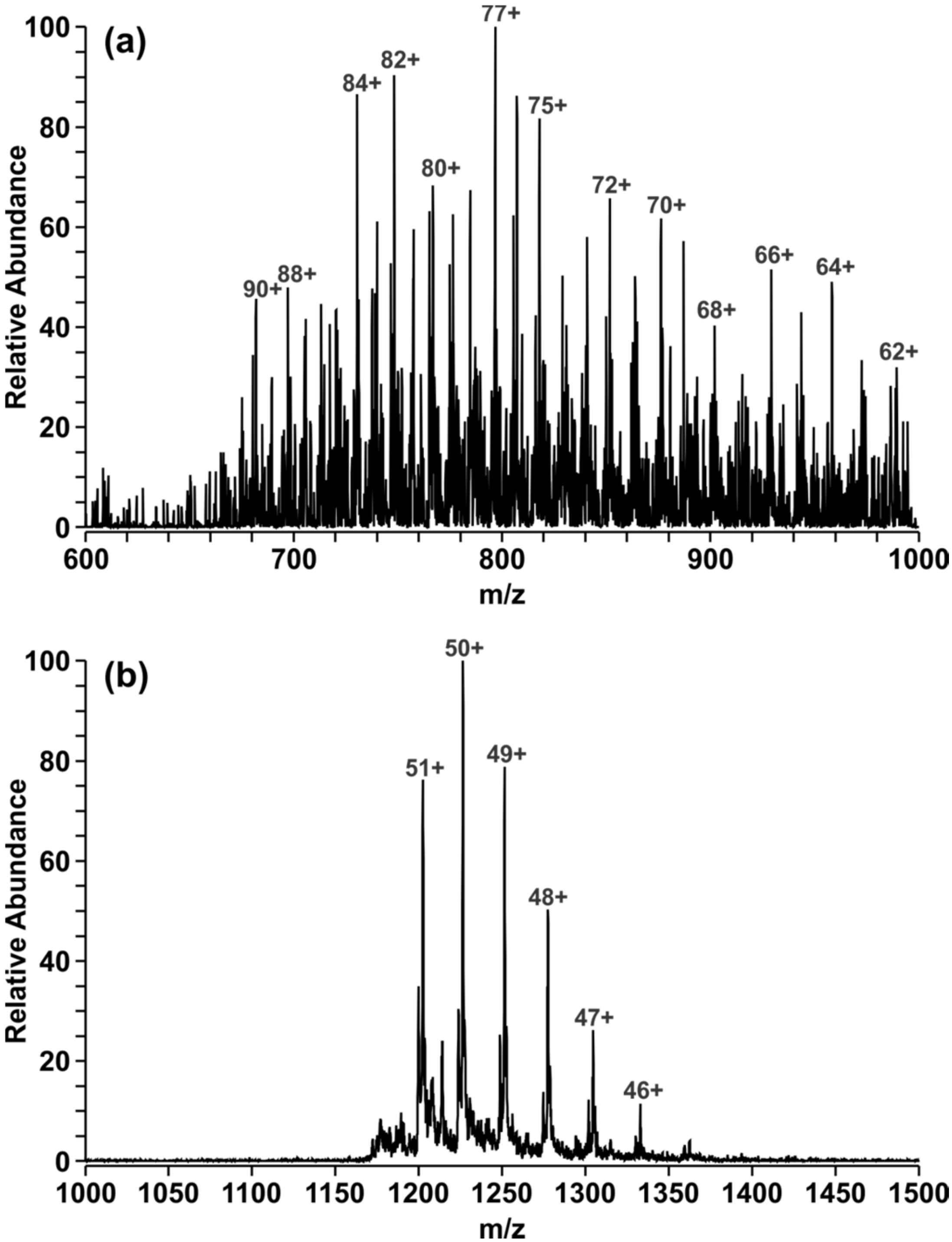

Supplemental Figure 4. (**a**) 400 Th isolation ion trap MS^2 spectrum depicting the +ESI charge state distribution of the 30S ribosomal protein S1 (61 kDa). (**b**) Subsequent ion trap IIPT/PIP MS^2 spectrum.
